# Investigating the Complexity of Multidimensional Symptom Experiences in Patients With Cancer: Systematic Review of the Network Analysis Approach

**DOI:** 10.2196/66087

**Published:** 2025-07-09

**Authors:** Vincent Richard, Allison Gilbert, Emanuela Pizzolla, Giovanni Briganti

**Affiliations:** 1 Department of Computational Medicine and Neuropsychiatry Faculty of Medicine University of Mons Mons Belgium; 2 Department of Medical Oncology CHU HELORA Hôpital de MONS, site Kennedy Mons Belgium; 3 Department of Neurosciences, Biomedicine and Movement Sciences University of Verona Verona Italy

**Keywords:** network analysis, symptoms, cancer patients, systematic review, cancer treatment, symptom management

## Abstract

**Background:**

Advances in therapies have significantly improved the outcomes of patients with cancer. However, multidimensional symptoms negatively impact patients’ quality of life. Traditional symptom analysis methods fail to capture the dynamic and interactive nature of these symptoms, limiting progress in supportive care. Network analysis (NA) is a promising method to evaluate complex medical situations.

**Objective:**

We performed a systematic review to explore NA’s contribution to understanding the complexity of symptom experiences in patients with cancer.

**Methods:**

The research question was as follows: “In patients with cancer (population), what is the contribution of NA (intervention) to understanding the complexity of multidimensional symptom experiences (outcome)?” The keywords “network analysis” AND “symptoms” AND “cancer survivors” OR “cancer patients” were searched in MEDLINE, Embase, Google Scholar, and Scopus between 2010 and 2024. Citations were extracted using Covidence software. Two reviewers independently screened the articles and resolved inclusion disagreements through consensus. Data were synthetized, and results have been narratively described. Bias analysis was performed using the Methodological Index for Non-Randomized Studies tool.

**Results:**

Among 764 articles initially identified, 22 were included. Studies evaluated mixed solid tumors (n=10), digestive tract cancers (n=4), breast cancer (n=3), head and neck cancer (n=2), gliomas (n=2), and mixed solid and hematological cancers (n=1). Twelve studies used general symptom assessment tools, whereas 10 focused on neuropsychological symptoms. Moreover, 1 study evaluated symptoms at diagnosis, 1 evaluated them during curative radiotherapy, 4 evaluated them during the perioperative period, 5 evaluated them during chemotherapy, 4 evaluated them during ongoing cancer therapies, and 7 evaluated them after acute treatments. Among these, 3 evaluated the longitudinal changes in symptom networks across chemotherapy cycles, and 1 evaluated changes during radiotherapy. Three studies investigated the associations between symptoms and biological parameters. Several NA approaches were used: network visualization (n=1), Bayesian network (n=1), pairwise Markov random field and IsingFit method (n=1), unregularized Gaussian graphical model (n=2), regularized partial correlation network (n=6), network visualization and community NA (n=1), network visualization and Walktrap algorithm (n=1), undirected network model with the Fruchterman-Reingold and edge-betweenness approaches (n=4), biased correlation and concise pattern diagram (n=1), extended Bayesian information criterion graphical LASSO method (n=3), cross-lagged panel network (n=1), and unspecified NA (n=3). Psychological symptoms, particularly anxiety, depression, and distress, were frequently identified as central and stably interconnected. Fatigue consistently emerged as a core symptom, closely linked to sleep disturbances, cognitive impairment, and emotional distress. Associations between symptoms and inflammatory biomarkers (eg, interleukin-6, C-reactive protein, and tumor necrosis factor-α) suggest a biological basis for symptom interconnectivity.

**Conclusions:**

NA consistently identified core symptoms, particularly psychological symptoms and fatigue, and associations with inflammatory biomarkers. NA may deepen the understanding of symptom interconnectivity and guide more effective interventions. However, further longitudinal homogeneous studies using standardized methodologies are needed.

## Introduction

The global burden of cancer is continuously increasing, with Europe accounting for one-fifth of the total cancer cases and cancer deaths [1]. Over the past 2 decades, advances in multidisciplinary management and tailored drug therapies have significantly improved treatment outcomes, offering potential cures or long-term remission and leading to the concept of cancer survivorship [2,3]. However, despite these medical advancements, many patients with cancer continue to experience persistent and complex symptoms resulting from both the disease and its treatments, negatively affecting their quality of life (QoL) for years after diagnosis [4,5]. New treatment opportunities provided by cancer research are often paired with unpleasant side effects, such as those observed with recent advances in immunotherapy, highlighting the need for a deeper understanding of symptom interactions to improve symptom management strategies [6,7].

Traditional approaches to symptom analysis, such as the symptom cluster approach [8,9], have sought to identify groups of co-occurring symptoms that share common mechanisms and clinical outcomes [10-12]. However, the symptom cluster approach has faced criticism due to its reliance on statistical grouping techniques that do not fully capture the dynamic relationships between symptoms and clusters [11,13-15]. Specifically, it lacks the ability to assess direct interactions within or between symptom clusters and does not account for causal relationships between symptoms [13,14]. These limitations have prompted researchers to explore network analysis (NA) as a novel methodological framework for studying symptom complexity [16,17]. NA, originally developed in mathematics and graph theory, has gained traction in psychological and medical research for its ability to estimate complex patterns of relationships and to reveal core features of mental disorders [18,19]. This approach grants a new ontological view on mental diseases, conceiving them as complex systems of components, which are maintained by mutual relationships between them, without the need to identify causal latent variables [17,19,20].

This network-based approach differs fundamentally from traditional models by conceptualizing diseases as interconnected systems rather than relying on predefined diagnostic categories [18].

In cancer research, NA offers a powerful framework for understanding symptom interactions, identifying core symptoms, and refining symptom management strategies. This approach could enable clinicians to develop targeted interventions, prioritizing symptoms that have the highest impact on patients’ QoL, which can ultimately enhance patient care [21]. In the study by Kossakowski et al [21], NA was used to analyze data related to health-related QoL in both a healthy population and patients with cancer, showing that maintaining daily routines and work activities could prevent symptom-related vicious cycles. Their findings emphasized the importance of psychosocial interventions in cancer treatment strategies [21].

Beyond symptom management, NA also holds promise for uncovering the underlying biological mechanisms driving symptom progression [22]. By integrating biological markers into symptom networks, this approach could provide new insights into pathophysiological pathways, offering opportunities for more biologically informed therapeutic strategies.

Kosvyra et al [22] explored the application of NA in the study of the biological data of patients with cancer, highlighting a significant gap in multiomics and predictive analyses, which limits the integration of biological mechanisms into symptom network research [22].

Despite promising findings, the application of NA in cancer symptom research remains fragmented, with existing studies often limited by sample heterogeneity, varied methodologies, and a lack of integration with biological data and therapeutic interventions. In this systematic review, we propose to investigate this complex and heterogeneous literature with a precise research question focusing on the contribution of NA in understanding the symptom experience of patients with cancer. The results will be detailed, and we will discuss methodological approaches used in existing studies, including differences in network construction and analysis; identify knowledge gaps; and propose future research directions.

By critically evaluating the existing literature, this review provides the first comprehensive assessment of the role of NA in understanding cancer symptomatology, emphasizing its potential to refine symptom management and enhance patient outcomes.

## Methods

### NA Approach

**Figure 1:**
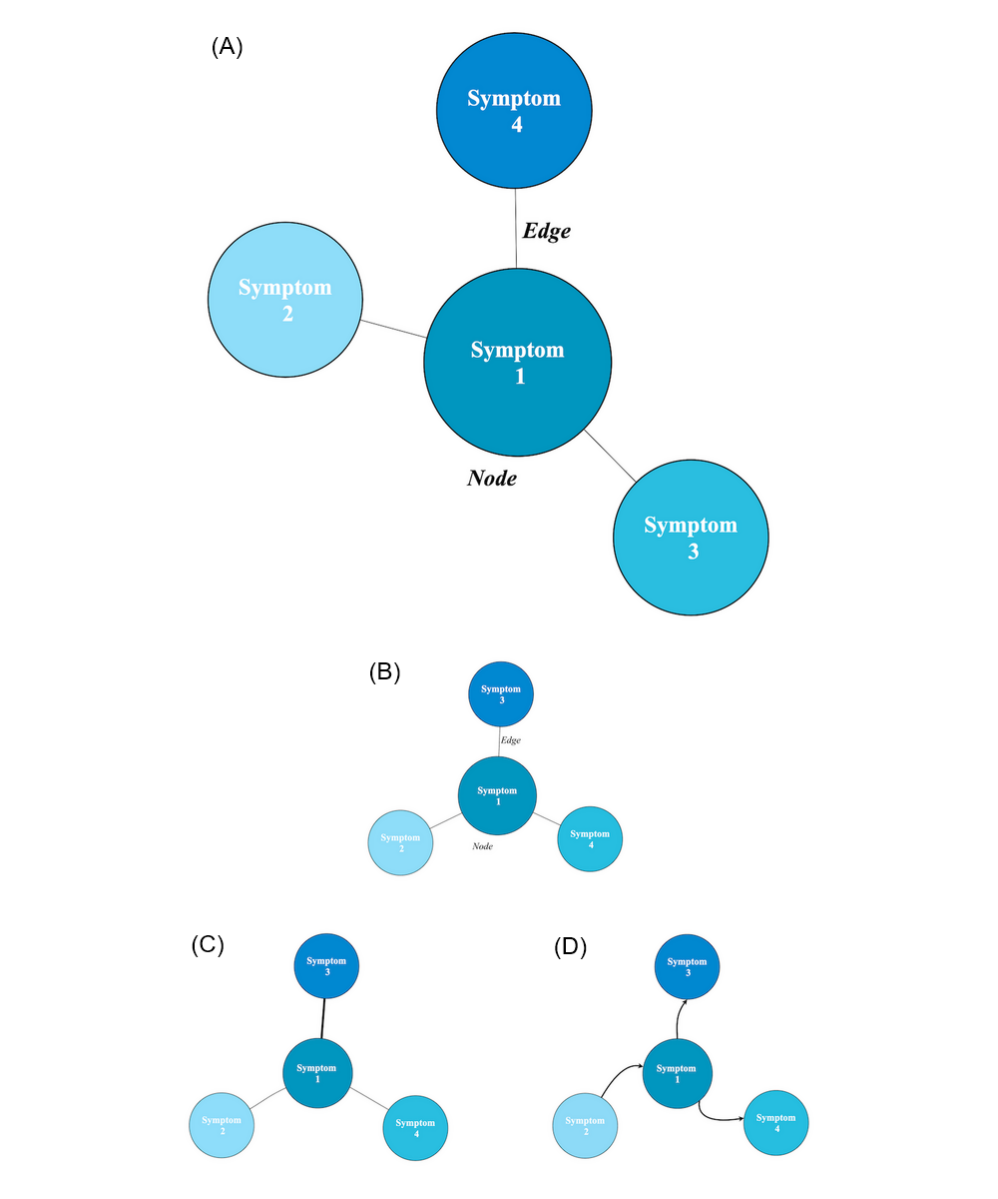
(A) Representation of 3 nodes (symptoms) with their relationships (edges). Networks: (B) unweighted, undirected network; (C) weighted network; and (D) cyclic or acyclic directed network.

A network is a set of nodes (variables) and a set of edges (statistical relationships) connecting the nodes [19]. In the medicine field, nodes are symptoms, and a network is a graphic representation of the complex association observable between symptoms. Several types of networks have been developed: *directed networks* (cyclic or acyclic), in which the direction of the edges is determined; *undirected networks*, in which the direction of the edges is unknown [19]; *weighted networks*, in which the weight of the edges is represented by their thickness and can represent a positive association or negative association; and *unweighted networks*, in which the edges either exist or not, and if they exist, they all have the same importance [18]. The classical structures of networks are represented in Figure 1.

NA has to follow a precise methodology: collect the data of interest (from cross-sectional, longitudinal, or panel data studies), construct the network, describe it, and analyze its stability [18,19]. The choice of the NA method influences network structure, the relationships captured, and the assumptions imposed on the data [20].

Once constructed, the structures of the network have to be analyzed in terms of its properties: what is the importance of nodes, is the global structure dense, and are the nodes isolated? These properties are described in terms of centrality (degree, node strength, closeness, betweenness, and clustering) [18]. Finally, the network accuracy has to be evaluated [19].

Pairwise Markov random fields and directed acyclic graphs are the most used methods in the psychopathological sciences [18]. Pairwise Markov random fields (Ising model and Gaussian graphical model) involve undirected models used to represent conditional dependence or independence between pairs of variables and are constructed using local conditional probability distributions [18]. The presence of an edge between 2 nodes indicates that they are conditionally dependent, and the absence of an edge indicates that they are conditionally independent. However, they do not explain model causal relationships. In contrast, directed acyclic graphs represent causal relationships, mapping directed interactions between symptoms without relying on probability distributions [18]. A comparative summary of these models is provided in Table 1.

**Table 1:** Comparison between pairwise Markov random fields and directed acyclic graphs.

Variable	Pairwise Markov random field	Directed acyclic graph
Graph type	Undirected graph	Directed acyclic graph
Edge interpretation	Encodes conditional dependencies between variables	Represents causal relationships between variables
Edge direction	No direction (edges are bidirectional)	Directed edges (A → B means A influences B)
Conditional independence	An edge’s presence or absence represents conditional dependence or independence	Uses d-separation to determine conditional independence
Causality	Does not assume causal relationships	Explicitly models cause-and-effect relationships
Loops/cycles	Can contain cycles	Acyclic (no feedback loops allowed)
Factorization of probability	Factorizes the joint distribution using local conditional distributions	Uses the chain rule to express joint probability based on parent-child relationships
Mathematical representation	Typically modeled using local Markov properties	Follows Bayes’ theorem to express probabilities
Common models	Ising model, Gaussian graphical model, and mixed graphical model	Bayesian network and structural equation model (SEM)
Handling of latent variables	Typically does not incorporate latent variables directly	Can explicitly include latent variables
Parameter estimation	Uses maximum likelihood estimation (MLE) or regularization techniques (eg, LASSO)	Parameters estimated using MLE, Bayesian inference, or SEM methods

### Research Question and Design

The research question was structured using the specialized PICO (population, intervention, comparator, outcome) framework. The final research question was as follows: “Considering patients with cancer (population), what is the contribution of the NA approach (intervention) to the understanding of the complexity of multidimensional symptom experiences (outcome)?” The PRISMA (Preferred Reporting Items for Systematic Reviews and Meta-Analyses) checklist was used to structure the report (Multimedia Appendix 1).

### Search Strategy

We systematically searched the following databases: PubMed (MEDLINE), Embase, Scopus, and Google Scholar. The search strategy was developed by the authors using a combination of medical subject headings, EMTREE thesaurus terms, and free-text keywords informed by an initial scoping review of the literature. No librarian or information specialist was consulted.

The search combined the terms “network analysis,” “symptoms,” and (“cancer patients” or “cancer survivors”) using Boolean operators. For multiword terms, quotation marks were used where appropriate (eg, “network analysis”). Filters were applied to limit results to studies on human adults published in English between 2010 and February 2024. A full description of the search strings and filters applied in each database is available in Multimedia Appendix 2.

References retrieved from the databases were imported into Covidence systematic review software, which automatically identified and removed duplicates. Additional references were identified through manual handsearching of Google Scholar.

### Selection Criteria

To be included in the review, the articles had to evaluate symptoms or symptom clusters in adult patients with cancer via an NA approach, either at diagnosis or during acute cancer treatment, long-lasting adjuvant therapy, and follow-up alone. To maintain some disease homogeneity, studies focusing on hematological patients alone were excluded, although those with mixed patient populations, solid tumors, or hematological cancers were admissible. Given that this review focuses on symptoms, articles evaluating QoL, coping strategies, or symptom-targeted interventions alone were excluded. Reviews or meta-analyses were also excluded. Eligible articles had to be written in English. This systematic review was not registered.

### Study Selection

The reference management software Covidence was used to export citations from database searches. Two reviewers (VR and AG) independently screened the titles and abstracts, and full-text screening was performed by both reviewers. Disagreements on inclusion were resolved through consensus.

### Data Extraction

A predefined extraction form was developed for data extraction. The process was performed by one reviewer (VR) and verified by a second reviewer (AG). Data were synthesized regarding different parameters: design of the study, main purpose of the study, sample size, cancer type, time of symptom assessment, tools used for symptom assessment and measures, NA methods, and main findings of the NA. The results are narratively described.

### Bias Analysis

The methodological quality and risk of bias of the included studies were assessed using the Methodological Index for Non-Randomized Studies (MINORS) tool. This validated instrument was chosen as it is specifically designed to assess the methodological quality of nonrandomized surgical studies, whether comparative or noncomparative, and has been adapted for use in systematic reviews across various medical fields [23].

The MINORS tool evaluates studies across 12 items: 8 items for noncomparative studies and an additional 4 items for comparative studies. Each item is scored as 0 (not reported), 1 (reported but inadequate), or 2 (reported and adequate). For noncomparative studies, the global ideal score is 16, while for comparative studies, it is 24. Two reviewers (EP and GB) independently conducted the bias assessment, with disagreements resolved through discussion until consensus was reached.

The evaluation criteria included clearly stated aims, consecutive patient inclusion, prospective data collection, appropriate endpoints, unbiased outcome assessment, appropriate follow-up period, loss to follow-up analysis, and prospective calculation of study size. For comparative studies, additional criteria included adequate control group selection, contemporary groups, baseline equivalence, and adequate statistical analysis.

## Results

### Search Results

A total of 764 articles were initially identified through searches across 4 literature databases. After title and abstract screening, 677 articles were excluded. Of the 39 full-text articles assessed for eligibility, 17 were excluded (9 due to the use of the wrong intervention and 8 due to an inappropriate study design). Ultimately, 22 studies were included in this review, comprising a cumulative total of 20,393 participants.

**Figure 2:**
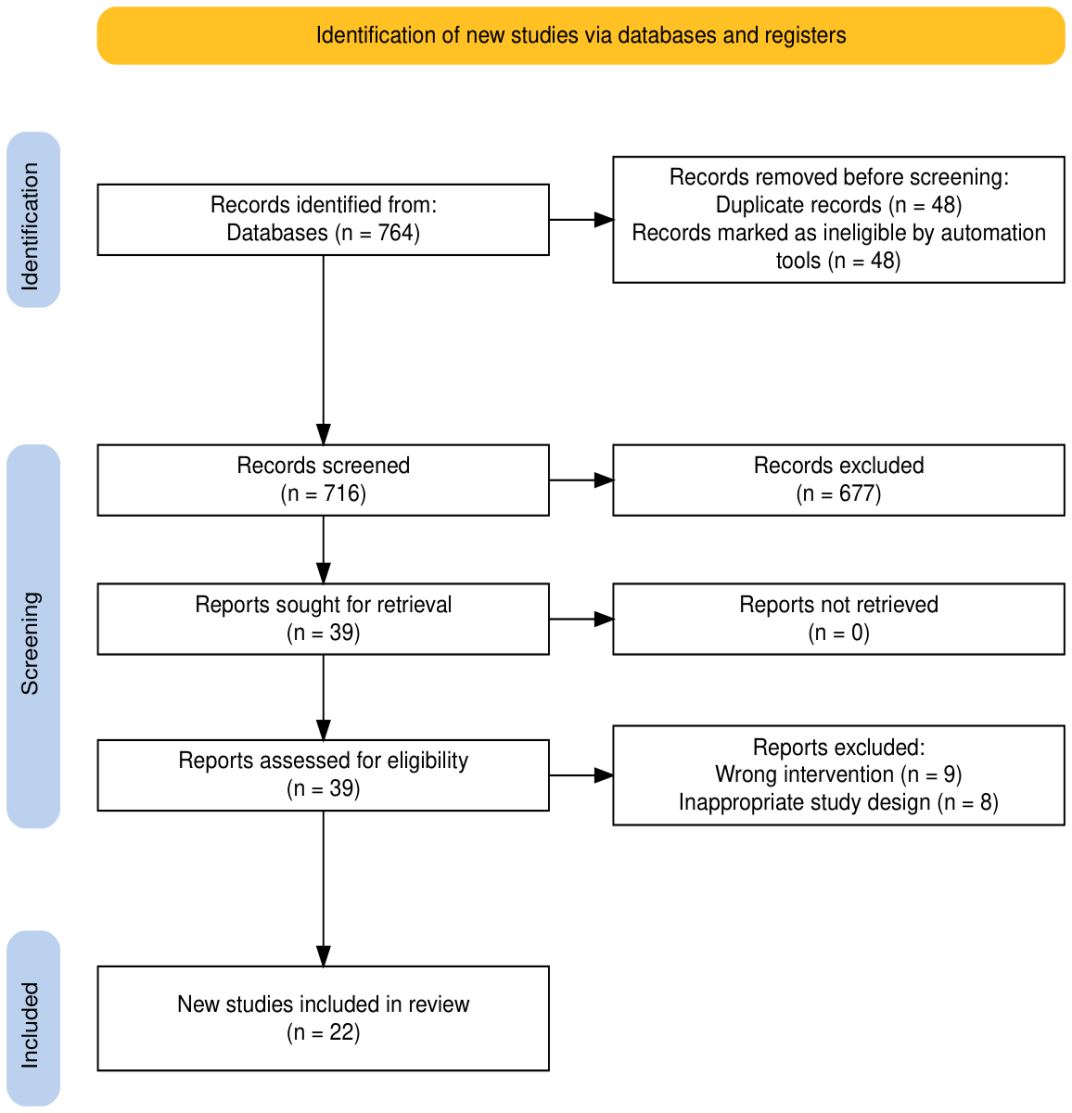
PRISMA 2020 flow diagram depicting the identification, screening, and inclusion process for studies in the systematic review.

The complete PRISMA flow diagram is presented in Figure 2. The diagram was generated using the PRISMA Flow Diagram Tool [24].

### Characteristics of the Included Studies

All characteristics of the selected studies are summarized in the subsections below and detailed in Table 2.

**Table 2:** Selected studies for the review.

Study and country	Study design	Main purposes	Sample description (including cancer type^a^) and assessment period	Symptom assessment: tools^b^ and biomarkers^c^
Bhavnani et al [25], 2010; United States	Secondary analysis of a randomized trial (2007); Longitudinal	Explore symptom co-occurrence and overlap patterns using network analysis. Explore quantitative measures to analyze symptom co-occurrence and overlap (observed patterns).	N=665 (463 female); Age: 21 years or older; Cancer type: solid tumors 94%, NHK 6%; Period: during CT^d^	Tools: MDASI (18 symptoms)
Xu et al [26], 2018; United States	Secondary analysis of a NIH^e^-funded study (2004-2010); Longitudinal	Investigate how depression, fatigue, and sleep interactions affect cognition and QoL^f^ during CT.	N=74 (74 female); Age: 51.8 (SD 9.5) years; Cancer type: BC; Period: pre-CT, post-CT, and 1 year after	Tools: PSQI (sleep quality), MFSI-SF (fatigue), CES-D (mood), FACT-B (quality of life), FOSQ (functional outcomes of sleepiness), and NP (cognition)
Papachristou et al [27], 2019; United States	Secondary analysis part of a longitudinal study	Evaluate the occurrence, severity, and distress of 38 cancer symptoms. Compare symptom networks based on occurrence, severity, and distress.	N=1328 (1032 female); Age: 57.2 (SD 12.4) years; Cancer type: BC 40.2%, GI 30.7%, GYN 17.3%, LC 13.2%; Period: during CT	Tools: mMSAS (38 symptoms)
Hartung et al [28], 2019; Germany	Cross-sectional from 2 studies: cross-sectional prospective patients with cancer; survey control general population	Compare depressive symptom severity, frequency, and networks between patients with cancer and the general population.	Study 1: N=4020 patients with cancer (2050 female); Age: 58 (SD 11) years Study 2: N=4020 individuals from the general population (2050 female); Age: 55 (SD 15) years Cancer type: BC 22.54%, PC 15.85%, CR 12.69%, and others 15.47%; Period: 14 months after diagnosis (mean)	Tools: PHQ-9 (depressive symptoms)
Schellekens et al [29], 2020; The Netherlands	Cross-sectional	Examine relationships among symptoms and psychosocial risk or protective factors.	N=342 (264 female); Age: 51.35 (SD 10.62) years; Cancer type: BC 45.6%, metastatic 36.8%; Period: ongoing treatments.	Tools: CIS-FS (fatigue), CES-D (depressive and anxiety symptoms), HDI (well-being), RSCL (physical symptoms), GSBQ (social withdrawal), ICQ (illness cognition), GAS (goal engagement), and WGS (partner support)
de Rooij et al [30], 2021; The Netherlands	Secondary analysis from the PROFILES registry and NCR^g^; Cross-sectional survey data	Identify symptom clustering across cancer types using network modeling.	N=1330 (835 female); Age: 61 (SD 15) years; Cancer type: BC 14.29%, CR 14.29%, Ov 14.29%, Thy 14.29%, HK 14.29%, NHK 14.29%, and CLL 14.29%; Period: years after diagnosis	Tools: EORTC-QLQ-C30 (30 symptoms; emotional and cognitive functioning scales)
Rha and Lee [31], 2021; Korea	Secondary data analysis from the SMILE RCT^h^; Longitudinal	Identify stable symptom clusters and their interrelationships across treatment cycles.	N=249 (184 female); Age: 51.89 (SD 9.75) years; Cancer type: BC 60.3%, GC 33.3%, and LC 6.4%; Period: across CT cycles	Tools: assessment of 20 symptoms, including 12 core symptoms, with a numerical rating scale (0-10)
Shim et al [32], 2021; Korea	Longitudinal	Study physical or psychological symptoms and QoL changes before or after gastric surgery.	N=256 (92 female); Age: 62.41 (SD 10.72) years; Cancer type: GC; Period: before and 1 week and 3-6 months after surgery	Tools: K-MDASI (Korean version; 13 symptoms), K-HADS (Korean version; depressive and anxiety symptoms), and FACT-Ga (QoL)
Henneghan et al [33], 2021; United States	Cross-sectional	Visualize symptom-cytokine networks and evaluate centrality in BC survivors.	N=66 (66 female); Age: 48.44 (SD 8.73) years; Cancer type: BC; Period: after adjuvant CT (6 months-10 years)	Tools: PCI-total (cognition), UCLA Loneliness Scale (loneliness), Perceived Stress Scale (stress), PROMIS (fatigue, anxiety, depression), PSQI (sleep quality), and Epworth Sleepiness Scale (daytime sleepiness); Biomarkers: 13 cytokines (TNF-α, GM-CSF, INF-γ, IL-2, IL-1b, IL-5, IL-7, IL-8, IL-10, IL-13, IL-6, IL-2, and IL-4)
Kalantari et al [34], 2022; United Kingdom	Longitudinal	Analyze changes in symptom clusters across treatment time points.	N=987 (779 female); Age: 56.9 (SD 12) years; Cancer type: BC 41.3%, GI 29.8%, GYN 17.7%, and LC 11.2%; Period: across 2 CT cycles, 6 time points	Tools: mMSAS (38 symptoms)
Lin et al [35], 2022; United States	Longitudinal	Examine temporal networks of psychoneurological symptoms.	N=172 (45 female); Age: 59.8 (SD 9.9) years; Cancer type: HNC; Period: 4 times across radiotherapy	Tools: PHQ-8 (depressive symptoms), MFI (fatigue), PSQI (sleep quality), and PRO-CTCAE (cognitive dysfunction and pain)
Santoso et al [36], 2022; The Netherlands	Cross-sectional	Link 5 psychoneurological symptoms with stress biomarkers in newly diagnosed HNC.	Cohort 1 (complete data): N=264 (55 female); Age: 65 (SD 8.2) years Cohort 2 (incomplete data): N=475 (135 female); Age: 62 (SD 10.4) years Cancer type: HNC; Period: at diagnosis and before treatment	Tools: PSQI (sleep quality), HADS (depressive and anxiety symptoms), EORTC-QLQ-H&N35 (EORTC-oral pain-related symptoms), and MFI (fatigue); Biomarkers: cortisol saliva, serum CRP, IL-6, IL-10, and TNF-α
Zhu et al [37], 2022; China	Cross-sectional	Explore network structure and symptom centrality in cancer survivors.	N=1065 (712 female); Age: 65.00 (SD 11.42) years; Cancer type: BC 29.3%, GI 22.6%, HNK 14.74%, and LC 14.46%; Period: cancer treatments completed (years)	Tools: MDASI (18 symptoms)
Ji et al [38], 2023; China	Cross-sectional	Identify clusters and core symptoms after esophageal cancer surgery.	N=286 (114 female); Age: 55.5% 65 years or older; Cancer type: early esophageal; Period: early postoperative	Tools: MDASI-GI
Röttgering et al [39], 2023; The Netherlands	Retrospective; Secondary analysis of merged studies	Compare global strength between symptom networks to understand if symptoms are more tightly connected in different subgroups of patients.	N=256 (95 female); Age: mean 47 years; Cancer type: glioma; Period: pre- and postoperative	Tools: CIS-FS (fatigue), CES-D (depressive symptoms), MOS-cog (cognitive functioning), EORTC-BN-20 (EORTC brain-tumor-related symptoms), and SF-36 (HRQoL^i^)
Jing et al [40], 2023; China	Secondary data analysis from a cross-sectional study	Explore symptom networks in patients with BC under endocrine therapy.	N=613 (613 female); Age: 49.5 (SD 9.4) years; Cancer type: BC; Period: endocrine therapy after acute care	Tools: FACT-ES (19 items)
Li et al [41], 2023; China	Cross-sectional	Study links between symptoms and inflammatory biomarkers in glioma.	N=203 (102 female); Age: 54.10 (SD 14.1) years; Cancer type: glioma; Period: during treatments	Tools: HAMA-14 (anxiety), HAMD-24 (depressive symptoms), PSQI (sleep quality), MFI (fatigue), and numerical rating scale 0-10 (pain); Biomarkers: IL-1β, IL-6, IL-4, IL-10, CRP, and TNF-α
Wang et al [42], 2023; China	Cross-sectional	Identify core symptom clusters in patients with DC.	N=202 (58 female); Age: 66.01 (SD 8.97) years; Cancer type: DC; Period: ongoing therapies.	Tools: MDASI-GI
Teng et al [43], 2024; China	Cross-sectional	Map symptom clusters and central symptoms after CT in patients with LC.	N=512 (139 female); Age: 65.21 (SD 8.94) years; Cancer type: LC (advanced 68%).; Period: post-CT	Tools: mMSAS (32 symptoms)
Kuang et al [44], 2024; China	Secondary analysis; Cross-sectional	Compare symptom networks by survivorship groups in elderly patients with cancer.	N=485 (295 female); Age: 72.54 (SD 6.44) years; Cancer type: elderly patients with cancer; Period: after acute treatments	Tools: MDASI (18 symptoms)
Shang et al [45], 2024; China	Prospective	Track predictive interactions between symptoms over time.	N=230 (94 female); Age: 66.13 (SD 10.80) years; Cancer type: operable CR; Period: pre- and postsurgery	Tools: MDASI-GI
Gong et al [46], 2024; China	Cross-sectional survey	Explore demoralization symptom networks in female patients with cancer.	N=413 (413 female); Age: 54.01 (SD 10.35) years; Cancer type: BC 63.2%, GC 18.4%, DC 10.7%, and others 7.7%; Period: ongoing therapies	Tools: DS-MV

^a^The following cancer types were identified in the included studies: breast cancer (BC), chronic lymphocytic leukemia (CLL), colorectal cancer (CR), digestive cancer (DC), gastric cancer (GC), gastrointestinal cancer (GI), gynecological cancer (GYN), Hodgkin lymphoma (HK), head and neck cancer (HNC), lung cancer (LC), non-Hodgkin lymphoma (NHK), ovarian cancer (Ov), prostate cancer (PC), and thyroid cancer (Thy).

^b^The following assessment tools were used in the included studies: Center for Epidemiologic Studies Depression Scale (CES-D), Checklist Individual Strength–Fatigue Severity (CIS-FS), Demoralization Scale Mandarin version (DS-MV), European Organization for Research and Treatment of Cancer Quality of Life Core Questionnaire (EORTC-QLQ-C30), Functional Assessment of Cancer Therapy–Breast Cancer (FACT-B), Functional Assessment of Cancer Therapy–Endocrine Subscale (FACT-ES), Functional Assessment of Cancer Therapy–Gastric Cancer (FACT-Ga), Functional Outcomes of Sleepiness Questionnaire (FOSQ), Goal Adjustment Scale (GAS), Groningen Social Behavior Questionnaire (GSBQ), Hospital Anxiety and Depression Scale (HADS), Hamilton Anxiety Scale–14 items (HAMA-14), Hamilton Depression Scale–24 items (HAMD-24), health and disease inventory (HDI), illness cognitions questionnaire (ICQ), MD Anderson Symptom Inventory (MDASI), MDASI–gastrointestinal cancer version (MDASI-GI), Multidimensional Fatigue Symptom Inventory–Short Form (MFSI-SF), Modified Memorial Symptom Assessment Scale (mMSAS), Medical Outcomes Study Cognitive Functioning Scale (MOS-cog), neuropsychological test battery composite score (NP), Perceived Cognitive Impairment scale (PCI), Patient Health Questionnaire–8 items (PHQ-8), Patient Health Questionnaire–9 items (PHQ-9), Patient-Reported Outcomes version of the Common Terminology Criteria for Adverse Events (PRO-CTCAE), Patient-Reported Outcomes Measurement Information System (PROMIS), Pittsburgh Sleep Quality Index (PSQI), Rotterdam Symptom Checklist (RSCL), 36-item short-form survey (SF-36), and ways of giving support (WGS).

^c^The following biomarkers were evaluated in the included studies: C-reactive protein (CRP), granulocyte-macrophage colony-stimulating factor (GM-CSF), interleukin (IL), interferon gamma (INF-γ), and tumor necrosis factor alpha (TNF-α).

^d^CT: chemotherapy.

^e^NIH: National Institutes of Health.

^f^QoL: quality of life.

^g^NCR: Netherlands Cancer Registry.

^h^RCT: randomized controlled trial.

^i^HRQoL: health-related quality of life.

#### Time of Publication

The included studies were published between 2010 and 2024, with 18 out of the 22 studies published between 2021 and February 2024.

#### Sample Sizes

The sample sizes ranged from 66 patients [33] to 4020 patients [28].

#### Design of the Study

Of the 22 studies, 7 were based on secondary data analyses and 15 were based on primary data (5 longitudinal and 10 cross-sectional studies).

#### Types of Cancer

Among the studies, mixed solid tumor populations were evaluated in 10 studies, digestive tract cohorts were assessed in 4 studies, breast cancer was evaluated in 3 studies, and head and neck cancers and gliomas were assessed in 2 studies. One study investigated a mixed cohort of solid and hematological cancers.

#### Tools for Symptom Assessment and Measures

Symptoms were assessed via 2 classes of validated tools. Twelve studies used general symptom assessment tools, such as the MD Anderson Symptoms Inventory and cancer type versions (n=6), the Modified Memorial Symptom Assessment Scale (n=3), the European Organization for the Research and Treatment of Cancer Quality of Life Questionnaire (n=1), the Twenty Symptoms List (n=1), and the Functional Assessment of Cancer Therapy-Endocrine Subscale (n=1), whereas 10 studies focused on neuropsychological symptoms via tools such as the Patient Health Questionnaire-9 (n=1), the Perceived Cognitive Impairment scale (n=1), the Hospital Anxiety and Depression Scale (n=1), and 7 other tools.

#### Time of Symptom Assessment

Considering the timing of symptom assessments, only 1 study evaluated symptoms at diagnosis before any treatment [36], 1 evaluated symptoms during curative radiotherapy [28], 4 evaluated symptoms during the perioperative period [32,38,39,45], 5 evaluated symptoms during chemotherapy [25-27,31,34], 4 evaluated symptoms during ongoing cancer therapies [29,41,42,46], and 7 evaluated symptoms after acute treatments [28,30,33,37,40,43,44]. Among these, 3 studies evaluated the longitudinal changes in symptom networks across chemotherapy cycles [26,31,34] and 1 evaluated changes during radiotherapy sessions [35]. Three studies investigated the associations between symptoms and biological parameters [33,41,43].

#### NA Approach

Several NA approaches and models were used to perform the studies included in this review. Some studies used different models: network visualization (n=1), Bayesian network (n=1), pairwise Markov random field and IsingFit method (n=1), unregularized Gaussian graphical model (n=2), regularized partial correlation network (n=6), network visualization and community NA (n=1), network visualization and Walktrap algorithm (n=1), undirected network model with the Fruchterman-Reingold approach and edge-betweenness approach (n=4), biased correlation network and concise pattern network diagram (n=1), extended Bayesian information criterion graphical LASSO method (n=3), cross-lagged panel network (n=1), and unspecified NA (n=3).

### Main Findings

In the following sections, we delve into the 22 included studies on the basis of the time of symptom assessment: at diagnosis and during or after acute cancer treatment.

#### Symptom Networks at Diagnosis

Only 1 study [36] evaluated symptom networks at cancer diagnosis (head and neck tumor). The connections between neuropsychological symptom networks and serum cytokines were also investigated. Four nodes had the most important position in the network: fatigue, poor sleep quality, C-reactive protein (CRP), and interleukin (IL)-6.

#### Symptom Networks During Acute Cancer Treatments

##### Chemotherapy

Five studies identified and evaluated symptom networks during chemotherapy.

Bhavnani et al [25] used NA to evaluate symptom co-occurrence in oncology, with a cohort of 665 patients with mixed tumors. Inspired by the results of symptom cluster research and its limitations, they used networks to visually analyze how 18 symptoms co-occurred across patients. They described a strongly nested structure of symptom co-occurrence, offering a new approach to the complexity of symptoms in patients with cancer.

Papachristou et al [27] investigated the relationships among 38 common symptoms in a cohort of 1328 patients with cancer, at 1 evaluation time point. They reported that the connections between and among symptoms may differ depending on the symptom dimension used to create the network (occurrence, severity, and distress). They identified a psychological symptom cluster that was stable across all 3 dimensions. They offered perspectives on the use of the network theory to develop new models aiming at improving therapeutic interventions for patients with cancer.

Other authors have reported the need for new interventions in patients with cancer. Rha and Lee [31] investigated clusters and the evolution of symptom networks across chemotherapy cycles in mixed solid tumor populations. They reported stable symptom clusters and evolving networks depending on the evaluation time point and the type of cancer, and the most central symptom identified was fatigue. The authors argued for the development of interventions focusing on central symptoms.

Kalantari et al [34] investigated 38 symptoms in 987 patients with cancer and assessed 4 different cancer types across 2 cycles of chemotherapy. They identified 8 relatively stable symptom clusters.

Xu et al [26] evaluated neuropsychological symptoms and QoL in a newly diagnosed breast cancer population across several chemotherapy cycles. They applied Bayesian network methods to investigate the role of sleep, fatigue, and mood on cognition and QoL across and after chemotherapy. They revealed strong direct and indirect links among symptoms, cognitive performance, and QoL. Sleep quality was directly linked to cognitive performance with late chemotherapy cycles. The authors concluded that a better understanding of the interrelationships among those symptoms, QoL, and cognition could guide the design of further effective interventions [26].

##### Radiotherapy

Lin et al [35] evaluated psychoneurological symptoms during radiotherapy (4 times) in 172 patients with newly diagnosed head and neck cancer. Depression and fatigue were the 2 core symptoms identified. The network structure was relatively stable over the treatment time. As previously suggested by other authors, Lin et al [35] argued that identifying core symptoms represents an opportunity to decrease other co-occurring symptoms.

##### Perioperative Period

Four studies evaluated symptom networks during the perioperative period (3 digestive tract tumor populations and 1 glioma population).

Röttgering et al [39] evaluated patterns of associations among depression, cognition, brain tumor–related symptoms, and QoL in a population of 256 diffuse gliomas. They constructed 6 networks based on the presence or absence of 3 disease statuses (surgical, tumor grade, and fatigue). Fatigue severity, depression, and social functioning were nodes highly correlated across the 6 networks. The number of nodes in the nonfatigued network was lower than that in the fatigued network. They suggested the need for integrative symptom management and targeted fatigue as a priority.

Other authors have reported the need to target specific psychological symptoms to reduce other interconnected symptoms [32]. Indeed, Shim et al [32] evaluated associations between cancer-related physical and psychological symptoms and QoL, before and after intent-to-cure surgery in 256 patients with gastric cancer. Distress and sadness were the most central symptoms across all time points. They identified connections between emotional and physical well-being.

Ji et al [38] reported that multiple symptom clusters occurred in a cohort of 286 patients with early esophageal cancer who were surgically treated. Sadness and fatigue were the core symptoms.

Shang et al [45] conducted a prospective study among 230 patients with operable colorectal cancer and evaluated 18 symptoms before and after surgery. They described a stable network with disturbed sleep and distress, which are the most prevalent symptoms to be targeted.

##### Ongoing Treatments

Several cross-sectional studies have evaluated networks in mixed cancer patient cohorts or mixed treatments (surgery, radiotherapy, chemotherapy, and hormonal treatment).

Among the objectives of NA research, identifying risk and protective factors has been discussed as an interesting approach to help further treatment strategies in patients with cancer. In 342 treated patients with cancer seeking psychological care, Schellekens et al [29] investigated the interconnectedness of fatigue, depression, anxiety, and potential risk and protective factors (physical symptoms, social withdrawal, illness cognition, goal adjustment, and partner support). Depressed mood, loss of enjoyment, and worthlessness were central nodes. Fatigue, anxiety, and depression appear strongly interconnected. Acceptance of illness was centrally embedded in the networks.

Wang et al [42] conducted a study among 202 treated patients with digestive cancer and identified distress, disturbed sleep, poor appetite, and sadness as the most common symptoms. The psychoemotional symptom cluster was the core symptom cluster.

Gong et al [46] explored the core and bridge symptoms of demoralization in 420 treated female patients with cancer. Discouragement, a lack of self-worth, hopelessness, and vulnerability were identified as the core and bridge symptoms.

#### Symptom Networks After Acute Cancer Treatments

Seven studies investigated symptom networks after acute cancer treatments, ranging from 1 week to several years.

Teng et al [43] identified 4 symptom clusters with a high stability network in 512 patients with advanced lung cancer 1 week after chemotherapy cycles.

Jing et al [40] explored the relationship of endocrine therapy–related symptoms to identify the core symptoms in a population of 613 patients with breast cancer receiving adjuvant hormonal treatment (average duration: 3.6 years). Mood swings and irritability were the most prevalent symptoms, and loss of interest in sex and joint pains were the most severe symptoms. There were no significant differences in network structure or global strength across treatment types (aromatase inhibitors vs selective estrogen receptor modulators) [40].

Concerning depressive symptoms in patients with cancer, Hartung et al [28] compared the frequency and relationships of depressive symptoms between patients with cancer and those in the general population. Depressive symptoms were much more common in patients with cancer but were less closely related to each other. Individual depressive symptom patterns should be considered in individuals rather than in group analyses.

de Rooij et al [30] aimed to explore the full complexity of symptoms. In a study evaluating symptom clusters in 1330 survivors of 7 cancer types, they reported that fatigue was consistently the most central symptom in an identified cluster and should be targeted. They concluded that interrelated symptoms may share the same underlying pathophysiological mechanisms, offering opportunities for new reflections on treatment strategies [30].

Henneghan et al [33] explored symptom networks in 66 patients with breast cancer after adjuvant chemotherapy (6 months to 10 years) and reported that stress, loneliness, depressive symptoms, and fatigue co-occur rather than occur as individual symptoms.

Zhu et al [37] investigated the symptom network of multidimensional symptom experiences and explored centrality indices and density networks in a cohort of 1065 patients with cancer who survived. They demonstrated that fatigue was the most severe symptom and that the density of the “less than 5 years” network was significantly different from that of the longest survivorship network. Distress, sadness, and lack of appetite were the core symptoms.

Kuang et al [44] explored symptom networks in 483 elderly patients with cancer who survived. The most common and severe symptoms were fatigue, disturbed sleep, and difficulty remembering. The density network showed differences between “less than 5 years” and “more than 5 years” survival.

### Symptom Networks and Biological Parameters

A study by Santoso et al [36] examined potential connections between psychoneurological symptoms (poor sleep, anxiety, and fatigue) and biomarkers of stress (cortisol slope, CRP, IL-6, IL-10, and tumor necrosis factor-α) in more than 264 patients with newly diagnosed head and neck cancer before treatment. Four nodes had the most important position in the network (fatigue, poor sleep quality, CRP, and IL-6) and may play a role in the interconnections between symptoms and inflammatory conditions.

Henneghan et al [33] investigated different symptoms and 13 cytokines in 66 patients with breast cancer at least 6 months and up to 10 years after adjuvant chemotherapy. Node betweenness was the highest for perceived cognitive impairment and the IL-2 level. Two separate communities of nodes (symptoms and cytokines) within the network were revealed and connected by several edges. They concluded that perceived cognitive impairment, stress, loneliness, depressive symptoms, and fatigue co-occur and that cytokines may be involved in these biological pathways.

A study by Li et al [41] evaluated the interrelations between neuropsychological symptoms and inflammatory biomarkers in a cohort of 203 patients with glioma. Depression, anxiety, fatigue, IL-6, and tumor necrosis factor-α had higher strength centrality indices and were identified as the most central nodes within the symptom-biomarker networks.

### Methodological Quality and Risk of Bias

The methodological quality of the 22 included studies was evaluated using the MINORS tool, and the results are summarized in Table 3. Overall, studies demonstrated moderate to high methodological rigor. Many studies, including the studies by Bhavnani et al [25], Xu et al [26], and Shim et al [32], reported clear aims and appropriate statistical analyses. Several studies, such as the studies by Rha and Lee [31], Kalantari et al [34], and Lin et al [35], employed longitudinal designs, enhancing their capacity to assess symptom dynamics over time. Other studies, such as the studies by Papachristou et al [27] and de Rooij et al [30], used large, heterogeneous samples with comprehensive network models, though some lacked follow-up or sample size reporting. Comparative studies scored well across all 12 MINORS criteria (eg, [28,40,42]). In contrast, noncomparative studies were assessed on the first 8 criteria and showed greater variability (eg, [33,36,46]). No studies were excluded based on their MINORS scores, but rather, the risk of bias assessment informed our interpretation of the findings and provided essential context for understanding methodological strengths and limitations across the reviewed literature.

**Table 3:** Quality assessment of the included studies.

Study	Category															Key commentary
	CA^a^	CP^b^	PD^c^	AE^d^	UA^e^	FP^f^	Loss <5%^g^	SSC^h^	CG^i^	CoG^j^	GE^k^	SA^l^	NCS^m^	CS^n^		
Bhavnani et al [25], 2010	2	2	1	2	2	0	0	0	2	0	0	2	13	13	Innovative study using network analysis to show symptoms that follow a nested structure rather than distinct clusters; the main limitation was secondary data analysis.	
Xu et al [26], 2018	2	1	2	2	2	2	0	0	0	0	0	2	11	11	Sophisticated Bayesian network modeling study; found that sleep quality during chemotherapy was directly linked to cognitive performance in patients with breast cancer.	
Papachristou et al [27], 2019	2	1	2	2	2	0	0	1	0	2	2	2	10	14	Large sample study showing that symptom networks differ by the dimension assessed (occurrence).	
Hartung et al [28], 2019	2	1	1	2	2	0	0	2	2	2	2	2	10	16	Rigorous study with large matched samples showing that depressive symptoms were more frequent but less interconnected in patients with cancer; suggested that external factors drive symptoms.	
Schellekens et al [29], 2020	2	2	2	2	2	0	0	1	0	0	0	2	11	11	Robust preregistered analysis of distressed patients with cancer; identified depressed mood.	
de Rooij et al [30], 2021	2	1	0	2	2	0	0	2	1	2	1	2	10	15	Strong methodological study examining symptom clusters across 7 cancer types; identified fatigue as a consistently central symptom; limited by cross-sectional design.	
Rha and Lee [31], 2021	2	1	2	2	2	2	0	1	0	0	0	2	12	12	Strong longitudinal analysis identified 3 stable symptom clusters across chemotherapy cycles, with fatigue as the most central symptom across all time points.	
Shim et al [32], 2021	2	2	2	2	2	2	0	0	0	0	0	2	12	12	Prospective study highlighting the central role of distress and sadness across perioperative time points; psychological symptoms served as bridges connecting symptoms to quality of life.	
Henneghan et al [33], 2021	2	0	2	2	2	0	0	0	0	0	0	2	8	8	Innovative exploratory study examining symptom-cytokine networks in breast cancer survivors; identified IL-2 and cognitive impairment as central; limited by a small sample size.	
Kalantari et al [34], 2022	2	1	2	2	2	2	0	0	0	2	1	2	11	14	Rigorous longitudinal study identifying 8 symptom clusters across chemotherapy cycles; demonstrated the stability of core symptoms over time despite changing severity.	
Lin et al [35], 2022	2	1	2	2	2	2	0	1	0	2	1	2	12	15	Strong longitudinal study examining symptom networks in patients with head and neck cancer; identified depression and fatigue as core symptoms across time points.	
Santoso et al [36], 2022	2	2	2	2	2	0	0	0	0	0	0	2	10	10	Large sample study found poor sleep.	
Zhu et al [37], 2023	2	1	2	2	2	0	0	0	0	0	0	2	9	9	Large sample study found distress, sadness, and lack of appetite as core symptoms in cancer survivors; network density was higher in survivors <5 years vs >5 years.	
Ji et al [38], 2023	2	1	2	2	2	0	0	0	0	0	0	2	9	9	Identified symptom clusters in early recovery after esophageal cancer surgery, with sadness and fatigue as core symptoms; good sample size but convenience sampling.	
Röttgering et al [39], 2024	2	1	1	2	2	0	0	0	0	2	1	2	8	11	Found fatigue.	
Jing et al [40], 2023	2	1	1	2	2	0	0	1	0	2	1	2	9	14	Strong analysis of endocrine therapy–related symptoms in patients with breast cancer; identified emotional symptoms as central regardless of treatment type.	
Li et al [41], 2023	2	1	2	2	2	0	0	0	0	0	0	2	9	9	Examined symptom-biomarker interconnections in patients with glioma; found depression.	
Wang et al [42], 2025	2	1	2	2	2	0	0	2	0	0	0	2	11	11	Well-designed study that identified a psychoemotional symptom cluster as core in patients with digestive cancer; distress had the highest centrality and the strongest bridge effect.	
Teng et al [43], 2024	2	1	2	2	2	0	0	0	0	0	0	2	9	9	Large sample study that identified the sickness behavior symptom cluster as most central in postchemotherapy patients with lung cancer; feeling irritable was a core symptom.	
Kuang et al [44], 2024	2	1	1	2	2	0	0	0	0	2	1	2	8	11	Examined symptom networks in older adults with cancer; found vomiting.	
Shang et al [45], 2024	2	1	2	2	2	2	0	0	0	0	0	2	11	11	Innovative longitudinal study using cross-lagged panel network analysis; identified disturbed sleep during admission as a predictor of subsequent symptoms in patients with colorectal cancer.	
Gong et al [46], 2024	2	1	2	2	2	0	0	0	0	0	0	2	9	9	Identified key demoralization symptoms in Chinese female patients with cancer; strengths included prospective data collection; limited by convenience sampling.	

^a^CA: clear aim.

^b^CP: consecutive patients.

^c^PD: prospective data.

^d^AE: appropriate end points.

^e^UA: unbiased assessment.

^f^FP: follow-up period.

^g^Loss <5%: loss to follow-up <5%.

^h^SSC: sample size calculation.

^i^CG: control group.

^j^CoG: contemporary groups.

^k^GE: group equivalence.

^l^SA: statistical analysis.

^m^NCS: noncomparative score (/16).

^n^CS: comparative score (/24).

## Discussion

### NA and Current Knowledge

Patients with cancer experience a highly interconnected network of co-occurring symptoms, which arise from both the disease itself and its treatment, significantly affecting QoL [4,5]. Traditional symptom analysis methods have primarily relied on symptom clustering approaches that fail to capture the dynamic interplay and mutual reinforcement between symptoms [13,14,16]. This review highlights the growing application of NA in oncology, demonstrating its potential to redefine symptom management by shifting the focus from treating individual symptoms or clusters of symptoms to identifying core symptoms that exert a broader influence on the overall network. As Papachristou et al [27] suggested, the network theory could offer interesting perspectives for understanding and focusing on specific symptoms to implement new therapeutic interventions, subsequently improving the management of patients with cancer.

Across the 22 included studies, NA was applied at different cancer treatment stages to identify key symptom interconnections and potential intervention targets. One of the most consistent findings was the prominent role of psychological symptoms, particularly anxiety, depression, and distress, which formed stable and interconnected networks, especially during chemotherapy and long-term survivorship [27,37,40]. For instance, Papachristou et al [27] found that psychological symptoms, such as anxiety, depression, and distress, tend to form stable networks during chemotherapy, influencing overall symptom burden. The stability of these networks suggests that psychological symptoms play a central role in shaping cancer symptomatology, potentially exacerbating physical symptoms through stress-related mechanisms. In support of this assumption, previous studies that did not apply NA have suggested that psychological disorders can substantially worsen physical symptoms in patients with cancer [47,48]. A clear example is the study by Renna et al [47] showing that breast cancer survivors with a distress disorder may be particularly at risk for more physical symptoms and treatment, reducing their QoL and increasing the recurrence risk.

Another recurrent finding in our work was the identification of fatigue as a central symptom across all treatment phases, with strong interconnections to sleep disturbance, cognitive impairment, and emotional distress [30,31,35]. Prominent studies, such as those by Rha and Lee [31] and Lin et al [35], reported that fatigue and depression consistently emerge as core symptoms, suggesting that targeting these symptoms may alleviate multiple co-occurring symptoms. The widespread influence of fatigue highlights the importance of psychophysiological symptoms in cancer symptom monitoring, reinforcing the need for targeted interventions that address not only fatigue itself but also its cascading effects on other symptoms.

In addition, while most studies were purely descriptive, a subset integrated biological markers into NA models, revealing significant associations among fatigue, depression, sleep disturbances, and inflammatory biomarkers such as IL-6, CRP, and tumor necrosis factor-α [33,36,41]. These findings suggest a possible biological underpinning of symptom clustering, aligning with existing evidence that inflammatory pathways may contribute to cancer-related fatigue and neuropsychological symptoms [49,50]. However, the mechanistic links between inflammation and symptom networks remain unclear, warranting further investigation.

Collectively, these findings support the hypothesis that targeting core symptoms, particularly fatigue and psychological distress, may provide a more effective therapeutic approach than treating symptoms in isolation. Despite the promising insights provided by NA, the studies reviewed were primarily descriptive, limiting their immediate clinical applicability. Further studies, particularly longitudinal studies and interventional trials, are necessary to determine whether NA-informed symptom management strategies can improve patient outcomes and facilitate the integration of network-based approaches into clinical practice.

### Implications for Clinical Practice

From the clinician’s point of view, we strongly believe that the NA approach could lead to innovations in interventions for patients with cancer. We thus argue for more longitudinal design studies investigating homogeneous patient cohorts. Consensus is required on tools to measure symptoms, with preference for polyvalent assessment (somatic and psychological symptoms).

With respect to network types, we suggest the use of Bayesian networks as the primary approach, considering the potential implementation of their outcomes in artificial intelligence datasets and consequently their use in clinical settings, especially in health-risk prediction and the evaluation of specific therapeutic interventions.

Finally, we believe that more clinician involvement (medical oncology, radiotherapy, oncological surgery, supportive care, and palliative care) in this area of research is highly necessary.

### Limitations and Perspectives for This Research Area

While this review offers a comprehensive synthesis of the current literature on the use of NA in cancer symptomatology, with most articles published during the last 3 years [30-46], some limitations must be acknowledged. First, there was considerable heterogeneity among the included studies in terms of cancer types, patient populations, sample sizes, symptom assessment tools, and network modeling techniques. This variability limited the ability to make direct comparisons across studies and precluded meta-analytic synthesis. Additionally, the majority of studies employed cross-sectional and exploratory designs, which, although valuable for hypothesis generation, limit the capacity to infer causality or observe symptom network evolution over time*.* Only a small number of studies incorporated biological markers [33,36,41], and none examined the impact of NA-informed therapeutic interventions, which constrains the applicability of the current findings to clinical practice.

In addition, although many included studies examined psychological symptoms, such as anxiety and depression, relatively few explicitly assessed neurocognitive functioning, despite its well-documented vulnerability to cancer treatments [51]. For example, while some studies incorporated cognitive performance indicators (eg, [26,39]), a more systematic and targeted exploration of cancer-related cognitive impairment within network models remains lacking. This represents an important research gap, as cancer-related cognitive impairment is increasingly recognized as a major component of cancer survivorship [51].

These limitations reflect broader gaps in the field and point to important directions for future research. First, there is a pressing need for longitudinal studies that can track changes in symptom networks across different treatment phases and survivorship. Such designs would enable researchers to identify critical time points at which symptom interconnectivity shifts, potentially informing more precise and timely interventions*.* Moreover, future research should move beyond descriptive modeling to include interventional studies, particularly randomized controlled trials designed to test whether targeting core symptoms identified through NA leads to measurable improvements in symptom burden and QoL. For example, evidence from non-NA–based trials has shown beneficial effects of physical activity and mind-body interventions (eg, yoga and mindfulness) on neuropsychological symptoms in cancer populations [52,53]. Incorporating such interventions into future network-informed studies could provide valuable insights into how these therapies affect symptom interconnectivity.

Another key area for development is the integration of biological and physiological data into NA frameworks. The limited but promising evidence linking symptom networks with inflammatory markers (eg, IL-6, CRP, and tumor necrosis factor-α) suggests that incorporating physiological data, including genomic, proteomic, and metabolomic variables, could shed light on the underlying mechanisms driving symptom interactions [33,36,41]. This could, in turn, support the development of biologically informed, personalized treatment strategies.

Furthermore, to ensure greater consistency and comparability across future studies, standardization of methodological approaches is essential. This includes the use of validated and comprehensive symptom assessment tools, consistent time points for symptom evaluation, and transparent reporting of network construction and statistical parameters. The development of consensus guidelines for conducting and reporting NA in oncology would represent a valuable step toward building a more cohesive and interpretable body of research.

In addition to study design variability, methodological considerations inherent to NA approaches must also be acknowledged. Several included studies used different NA techniques, such as Gaussian graphical models or Bayesian networks, with varying assumptions, sparsity constraints, and estimation procedures. The sensitivity and specificity of these models in capturing symptom interconnections depend heavily on data quality, sample size, and statistical regularization methods [20]. Furthermore, the stability of centrality measures and the reproducibility of network structures were not systematically evaluated across studies, limiting conclusions about the robustness and generalizability of findings [54]. Greater methodological standardization and reporting transparency are needed to ensure the validity of symptom networks in oncology.

In summary, while current studies provide compelling evidence for the potential of NA to enhance symptom understanding in cancer care, addressing methodological limitations and expanding the scope of research are essential next steps.

### Conclusions

Cancer is a complex disease that causes significant disruption to both biological systems and overall health, leading to complex, interrelated symptom experiences in patients with cancer. This review highlights the growing application of NA as a valuable tool for understanding the complexity of cancer-related symptoms.

Across the included studies, NA consistently identified core symptoms, particularly psychological symptoms and fatigue, that appear central to patients’ experiences across treatment stages. These findings suggest that focusing on core symptom interconnectivity may offer more effective avenues for symptom management than traditional approaches targeting isolated symptoms.

While current research offers compelling evidence for the application of NA in cancer symptomatology, several methodological limitations persist. Future studies should focus on longitudinal designs that track symptom networks across different phases of cancer treatment and survivorship. Further research should also explore interventional approaches to determine whether NA-informed strategies can improve symptom management and enhance QoL. Integrating biological and physiological data into NA frameworks holds promise for developing personalized, biologically informed treatment strategies.

Standardization of methodological approaches, including validated symptom assessment tools, and transparent reporting of network construction are essential to strengthen the consistency and comparability of findings across studies. Ultimately, network-informed research can contribute to a deeper understanding of cancer symptom interconnectivity and lead to more effective and targeted interventions, improving outcomes for patients with cancer.

## Appendix

Multimedia Appendix 1 PRISMA checklist.

Multimedia Appendix 2 Detailed search strategies.

## Abbreviations

CRP: C-reactive protein
IL: interleukin
MINORS: Methodological Index for Non-Randomized Studies
NA: network analysis
PICO: population, intervention, comparator, outcome
PRISMA: Preferred Reporting Items for Systematic Reviews and Meta-Analyses
QoL: quality of life
