# Supplementary material for: Investigating the Complexity of Multidimensional Symptom Experiences in Patients With Cancer: Systematic Review of the Network Analysis Approach
Source: JMIR Cancer. 2025 Jul 9;11:e66087. doi: 10.2196/66087 (PMC12287671; doi:10.2196/66087)
Supplement: Multimedia Appendix 2 [file cancer_v11i1e66087_app2.docx]

**Multimedia Appendix 2.** Detailed search strategies.

**1. Databases Searched**

PubMed (Medline); Embase; Scopus; Google Scholar (manual handsearching)

**2. Search Period**

January 2010 to February 2024

**3. Filters Applied**

English language; Human subjects; Peer-reviewed articles only; Adults (excludes pediatric populations)

**4. Search Terms and Keywords**

The following keywords and subject headings (MeSH/EMTREE) were used in combination with Boolean operators:

Keywords*:* "network analysis"; "symptoms"; "cancer patients"; "cancer survivors"

Boolean Logic Applied*:* ("network analysis") AND ("symptoms") AND ("cancer patients" OR "cancer survivors")

**5. Database-Specific Strategies**

**Pubmed Search Strategy**

| **Search No.** | **Search Details** | **Results** |
| --- | --- | --- |
| 1 | "network analysis"[Title/Abstract] | 34,082 |
| 2 | "diagnosis"[MeSH Subheading] OR "diagnosis"[All Fields] OR "symptoms"[All Fields] OR "diagnosis"[MeSH Terms] OR "symptom"[All Fields] OR "symptom s"[All Fields] OR "symptomes"[All Fields] | 12,004,602 |
| 3 | "cancer patients"[Title/Abstract] | 239,902 |
| 4 | "cancer survivors"[Title/Abstract] | 24,088 |
| 5 | ("network analysis"[Title/Abstract] AND ("diagnosis"[MeSH Subheading] OR "diagnosis"[All Fields] OR "symptoms"[All Fields] OR "diagnosis"[MeSH Terms] OR "symptom"[All Fields] OR "symptom s"[All Fields] OR "symptomes"[All Fields]) AND ("cancer patients"[Title/Abstract] OR "cancer survivors"[Title/Abstract])) AND (humans[Filter]) | 188 |
| 6 | ("network analysis"[Title/Abstract] AND ("diagnosis"[MeSH Subheading] OR "diagnosis"[All Fields] OR "symptoms"[All Fields] OR "diagnosis"[MeSH Terms] OR "symptom"[All Fields] OR "symptom s"[All Fields] OR "symptomes"[All Fields]) AND ("cancer patients"[Title/Abstract] OR "cancer survivors"[Title/Abstract])) AND ((humans[Filter]) AND (2010:2024[pdat])) | 177 |

**Embase Search Strategy**

| **Search No.** | **Search Details** | **Results** |
| --- | --- | --- |
| 1 | 'network analysis'/exp OR 'network analysis' | 44186 |
| 2 | 'symptoms' | 1722181 |
| 3 | 'cancer patient' | 707828 |
| 4 | 'cancer survivor' | 39103 |
| 5 | #1 AND #2 | 2436 |
| 6 | #3 OR #4 | 738416 |
| 7 | #5 AND #6 | 49 |

**Scopus Search Strategy**

| **Search No.** | **Search Details** | **Results** |
| --- | --- | --- |
| 1 | TITLE-ABS-KEY ( network AND analysis ) | 1,722,830 |
| 2 | TITLE-ABS-KEY ( symptoms ) | 1,913,118 |
| 3 | TITLE-ABS-KEY ( cancer AND patients ) | 2,069,650 |
| 4 | TITLE-ABS-KEY ( cancer AND survivors ) | 60,578 |
| 5 | #1 AND #2 | 23,945 |
| 6 | #3 OR #4 | 2,089,907 |
| 7 | #5 AND #6 | 1,243 |
| 8 | ( ( TITLE-ABS-KEY ( network AND analysis ) ) AND ( TITLE-ABS-KEY ( symptoms ) ) ) AND ( ( TITLE-ABS-KEY ( cancerAND patients ) ) OR ( TITLE-ABS-KEY ( cancer AND survivors ) ) ) AND PUBYEAR > 2009 AND PUBYEAR < 2025 AND (LIMIT-TO ( SUBJAREA , "medi" ) OR LIMIT-TO ( SUBJAREA , "comp" ) OR LIMIT-TO ( SUBJAREA , "nurs" ) OR LIMIT-TO( SUBJAREA , "neur" ) OR LIMIT-TO ( SUBJAREA , "psyc" ) OR LIMIT-TO ( SUBJAREA , "phar" ) OR LIMIT-TO (SUBJAREA , "heal" ) ) AND ( LIMIT-TO ( DOCTYPE , "ar" ) OR LIMIT-TO ( DOCTYPE , "cp" ) ) AND ( LIMIT-TO (EXACTKEYWORD , "humans" ) ) AND ( LIMIT-TO ( LANGUAGE , "english" ) OR LIMIT-TO ( LANGUAGE , "french" ) ) | 530 |

**6. Reference Management**

All retrieved references were imported into Covidence, which automatically removed duplicates. The PRISMA flow diagram reflects this process.
